# Advanced interatrial block predicts ineffective cardioversion of atrial fibrillation: a FinCV2 cohort study

**DOI:** 10.1080/07853890.2021.1930139

**Published:** 2021-05-21

**Authors:** Arto Relander, Tapio Hellman, Tuija Vasankari, Ilpo Nuotio, Juhani K. E. Airaksinen, Tuomas Kiviniemi

**Affiliations:** a Heart Center, Turku University Hospital and University of Turku, Turku, Finland; b Department of Medicine, Turku University Hospital and University of Turku, Turku, Finland; c Division of Cardiovascular Medicine, Brigham and Women’s Hospital and Harvard Medical School, Boston, MA, USA

**Keywords:** Atrial fibrillation, cardioversion, electrocardiogram, interatrial block, P-wave, FinCV

## Abstract

**Aims:**

Rhythm control using electrical cardioversion (CV) is a common treatment strategy for patients with symptomatic atrial fibrillation (AF). To guide clinical decision making, we sought to assess if electrocardiographic interatrial blocks could predict CV failure or AF recurrence as the phenomenon is strongly associated with atrial arrhythmias.

**Methods:**

This study included 715 patients who underwent a CV for persistent AF lasting >48 h. P-wave duration and morphology were analyzed in post-procedure or the most recent sinus rhythm electrocardiograms and compared with rates of CV failure and AF recurrence within 30 days after CV as well as their combination (ineffective CV).

**Results:**

CV was unsuccessful in 63 out of 715 patients (8.8%) and AF recurred in 209 out of 652 (29.2%) patients within 30 days after CV. Overall, 272 (38.0%) CVs turned out ineffective. Advanced interatrial block (AIAB) defined as P-wave duration ≥120 ms and biphasic morphology in inferior leads (II, III and aVF) was diagnosed in 72 (10.1%) cases. AIAB was an independent predictor for CV failure (OR 4.51, 95%CI 1.76–11.56, *p* = .002), AF recurrence (OR 2.93, 95%CI 1.43–5.99, *p* = .003) and ineffective CV (OR 3.87, 95%CI 2.04–7.36, *p* < .001).

**Conclusion:**

AIAB predicted CV failure, AF recurrence as well as their composite. This study presents an easy electrocardiographic tool for the identification of patients with persistent AF who might not benefit from an elective CV in the future.

**KEY MESSAGES**
- Interatrial blocks are very common in patients with atrial fibrillation.
- Advanced interatrial block predicts ineffective cardioversion.

## Introduction

Sinus rhythm (SR) is often restored by cardioversion (CV) especially in symptomatic atrial fibrillation (AF) patients. Unfortunately, a significant proportion of CVs turn out to be ineffective: approximately 10–35% fail initially and 30–60% of AFs recur within a month [1–4]. Earlier studies have identified predictors of ineffective CV, namely female sex, young age (<65 years) and low (<60 ml/min) estimated glomerular filtration rate (eGFR) [2–4].

Small retrospective datasets suggest that electrocardiographic (ECG) markers, such as interatrial blocks predict AF recurrence [1,5–7], but information about CV failure or CV inefficacy overall is scarce. Interatrial block refers to a significant conduction defect between the atria. Atrial enlargement and fibrosis generally promote conduction delays, but usually, interatrial blocks exist when parts of the Bachmann (interatrial) bundle are impaired. Distinct block patterns were described by de Luna et al. [8,9] and can be easily recognized on a standard 12-lead ECG. Such alterations in electrical properties of the atria often precede rhythm disturbances and the electrocardiographic interatrial block has been associated with the development of AF and other atrial tachyarrhythmias [8–10]. This study sought to explore the role of interatrial blocks in predicting ineffective elective CV.

## Methods

The FinCV2 study (http://www.clinicaltrials.gov, identifier NCT02850679) is part of a wider protocol in progress to assess clinical challenges of AF in Western Finland [11,12]. Originally, the data in this multi-center retrospective cohort study were gathered manually from the patient records using a structured case report. Patients were identified using the ICD-10 code for AF (I48) and the NCSP (Nordic Classification of Surgical Procedures) procedure code for CV (TFP20). Patients undergoing an elective electrical CV for AF lasting >48 h in two university hospitals or two regional hospitals in Finland were eligible for the original FinCV2 study population. To be included in this sub-study patient ECGs had to be available at Turku university hospital via the electronic MUSE-ECG database. We also excluded patients with a history of catheter ablation for atrial fibrillation due to possible aberrations to the ECG.

The study received approval from the Medical Ethics Committee of the Hospital District of Southwest Finland and the ethics committee of the National Institute for Health and Welfare. The study conforms to the Declaration of Helsinki. Informed consent was not required because of the retrospective nature of the study. Data underlying this article is available on a reasonable request to the corresponding author.

**Figure 1.**
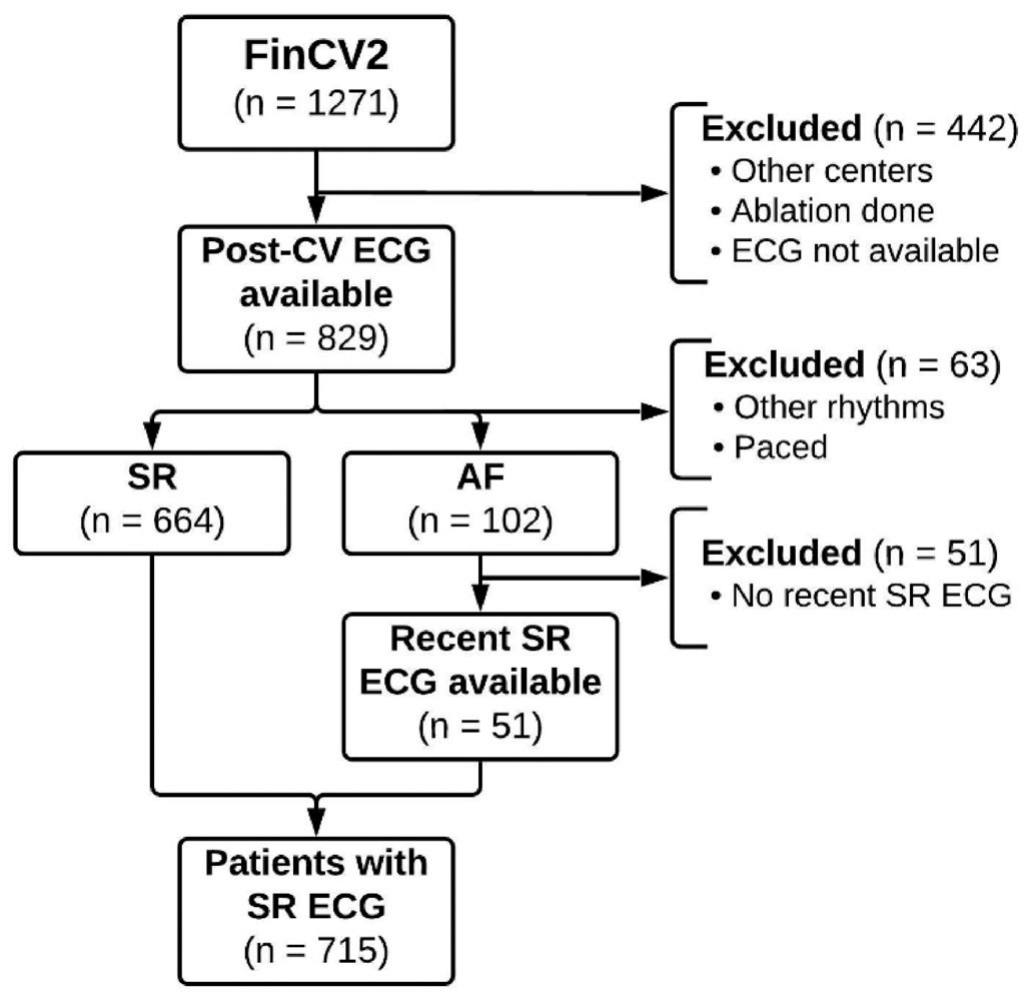
Number of patients who were initially screened, had their ECG examined and finally included in the study. AF: atrial fibrillation; CV: cardioversion; ECG: electrocardiogram; SR: sinus rhythm.

Flow chart of the study setting is presented in Figure 1. Overall, 1271 patients were initially screened, and their medical history was acquired from the electronic database for the original study. Patients from other centres (*n* = 392), with a history of catheter ablation (*n* = 16) or with bad quality or missing ECG (*n* = 34) were excluded, leaving 829 recordings immediately post-CV. Other than SR and AF recordings were discarded (*n* = 63). SR was restored in 664 CVs, while 102 remained in AF. Before hospital discharge 16 patients regressed to AF, however, an SR ECG was successfully recorded. Of those with a failed CV (AF in post-CV recording), the most recent ECG was obtained. We included recent recordings up to 60 months prior (*n* = 41) and up to 12 months post-CV (*n* = 10) in this study, the average time was 17 (standard deviation 17.5) months prior CV. The final study cohort comprised 715 ECGs of individual patients in SR.

Elective CVs were performed by an attending internist or cardiologist under general anaesthesia according to the current guidelines. ECGs were recorded and interpreted by the clinician prior to and after the procedure to confirm AF and determine the CV outcome, respectively. The CV energy and positioning of the defibrillator paddles (anterolateral or anteroposterior configuration) were left to the discretion of the attending clinician. Data on CV energy and defibrillator positioning is unavailable. CVs were performed using biphasic defibrillators after 2004. Transesophageal echocardiograms were not routinely performed if anticoagulating agents were used as prescribed for at least 3 weeks. Previous transthoracic echocardiogram data was available on 218 (30.5%) cases. Left atrium diameter measurements were collected. Mild left atrial enlargement was defined as diameters > 41 mm in males and >39 mm in females, moderate enlargement as diameters >47 mm and >43 mm, respectively.

### Electrocardiogram measurements

We used standard 12-lead ECGs with 50 mm/s recording speed and 10 mm/mV voltage gain. ECGs were interpreted by a single observer with the possibility to consult a second expert observer, when in doubt. Observers were blinded to the clinical data and CV outcomes. Measurements were made manually with 0.25 mm (5 ms, 0.025 mV) precision, always rounding down.

**Figure 2.**
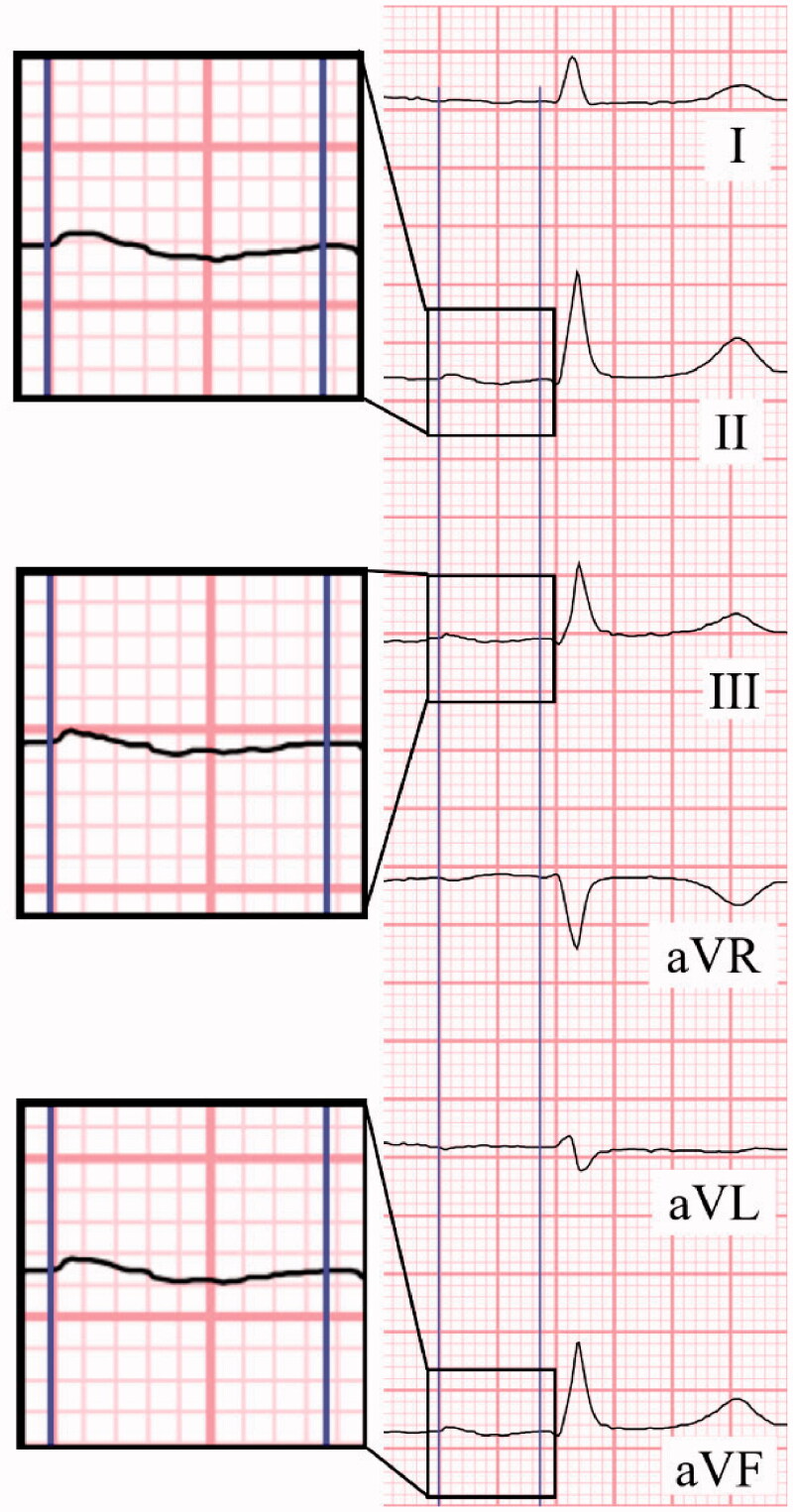
Determining P-wave duration and morphology. Earliest initiation and latest ending of the P-wave are identified through all the limb leads to determine the true duration of the atrial activation. Morphology is assessed from leads II, III and aVF, here a biphasic wave is seen in all inferior leads. A typical case of advanced interatrial block.

P-wave duration was measured identifying the earliest and latest detections in the limb leads. Morphology was considered biphasic if the P-wave had both positive and negative deviations (≥20 ms, −1 mm) from the baseline in the inferior leads (Figure 2). An elongated P-wave (≥120 ms) was categorized as a partial interatrial block (PIAB). The block was considered advanced (AIAB) when the P-wave clearly has a biphasic (+/−) morphology in all inferior leads (II, III, aVF) in addition to elongation. To be labelled biphasic the final part of the wave had to be at least 20 ms and 0.025 mV below the baseline.

We used the machine calculated duration for PR-interval and roughly verified the duration manually. A PR-interval of ≥200 ms was considered first-degree atrioventricular block (AV-block).In lead V1 P-wave terminal force (PTF) was calculated as the product of duration and voltage of the negative portion of the P-wave. Left ventricular hypertrophy was assessed with Cornell, modified Cornell, and Sokolow–Lyon criteria. Ventricular conduction issues included bundle branch blocks and their incomplete forms, fascicular blocks, and prolonged QRS-complexes without any specific morphology [13].

We analyzed additional 50 ECGs from random patients with a successful CV to rule out any changes in P-wave measurements that might be due to the post-CV atrial stunning phenomenon. The ECG database was searched for non-CV-related SR films within one year from the indexed CV. ECGs related to other cardiac procedures were also excluded.

### Clinical outcomes

The primary endpoint of the study was ineffective CV, the composite of CV failure and early AF recurrence (within 30 days follow-up). CV was considered successful if SR was maintained until hospital discharge. AF was confirmed by ECG or a pacemaker log to be reliably defined as recurrent AF. Safety outcomes included asystole (>5 s) or bradycardia (<40/min) after CV as well as thromboembolic complications and death during the 30 days follow-up.

### Statistical analysis

**Figure 3.**
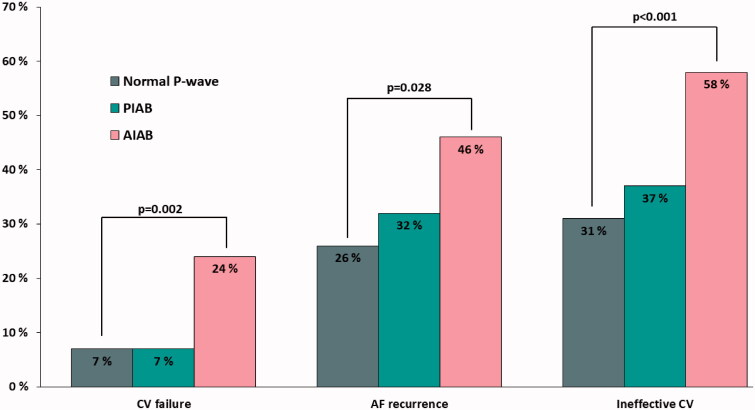
Incidence of CV failure, AF recurrence and the composite (ineffective CV) expressed by P-wave characteristics. AIAB: advanced interatrial block; AF: atrial fibrillation; CV: cardioversion; PIAB: partial interatrial block.

Statistical analyses were conducted with SPSS version 25.0 statistical software (SPSS, IBM SPSS Inc., Chicago, IL, USA) and R statistics software version 3.5.3 (R Foundation for Statistical Computing, Vienna, Austria). Continuous variables were reported as mean ± standard deviation if normally distributed, and as median (25th–75th percentiles) if they were skewed. Categorical variables were described as counts and percentages. Logistic regression, Pearson’s Chi-square, Fischer’s exact test, Wilcoxon rank sum test and Kruskal–Wallis test were used for univariable analyses, as appropriate. Cochran Armitage trend-test was used to test trends between multiple groups. Bonferroni method and Steel’s nonparametric multiple comparisons with control were applied in post-hoc testing when applicable in Table 1, Figure 3 and the results chapter. Relationships between continuous variables were studied with Pearson’s correlation. A multivariable logistic regression model was created to study how, both, PIAB and AIAB predict ineffective CV compared to the normal P-wave. Backward stepwise selection was used with variables with a *p*-value <.10 in the univariable analysis. This model was further used to assess predictors of CV failure and AF recurrence. A *p*-value <.05 was considered statistically significant.

**Table 1.** Baseline characteristics of patients with normal P-wave, partial interatrial block or advanced interatrial block.

	Normal P-wave (*n* = 119)	PIAB (*n* = 524)	AIAB (*n* = 72)	p*	p**
Age, years	64 (55, 70)	64 (57, 71)	70 (62, 77)	.849	<.001
Sex, female	29 (24.4)	86 (16.4)	25 (34.7)	.094	.276
CHA_2_DS_2_-VASc	1 (0, 3)	2 (1, 3)	2 (1, 4)	.219	<.001
CHA_2_DS_2_-VASc >1	59 (49.2)	288 (54.0)	51 (69.9)	.726	.014
Heart failure	13 (10.9)	97 (18.5)	10 (13.9)	.116	.999
Hypertension	49 (41.2)	274 (52.3)	42 (58.3)	.066	.050
eGFR <60 ml/min^a^	7 (12.1)	33 (13.1)	10 (28.6)	.999	.112
Diabetes	16 (13.4)	76 (14.5)	13 (18.1)	.999	.822
Prior stroke or TIA	6 (5.0)	32 (6.1)	10 (13.9)	.999	.112
Vascular disease	12 (10.1)	90 (17.2)	20 (27.8)	.140	.004
First AF episode	70 (58.8)	357 (68.1)	52 (72.2)	.134	.130
AF episode over 30 days^a^	43 (66.2)	175 (72.3)	30 (75.0)	.714	.776
AF history over 180 days^b^	45 (44.6)	173 (40.0)	20 (37.0)	.864	.794
Prior CV^c^	35 (31.5)	110 (21.9)	12 (17.6)	.072	.108
Left atrial diameter, mm^d^	44.5 (40, 48)	47 (42, 50)	44.5 (38, 47)	.041	.800
Mild enlargement	33 (68.8)	130 (82.3)	7 (58.3)	.132	.999
Moderate enlargement	16 (33.3)	71 (44.9)	4 (33.3)	.366	.999
Medication at CV^e^					
Verapamil	3 (2.6)	8 (1.5)	1 (1.4)	.866	.999
Digoxin	26 (22.2)	133 (25.5)	18 (25.0)	.999	.999
B-Blocker	97 (82.9)	423 (80.7)	59 (81.9)	.999	.999
Any antiarrhythmic	8 (6.8)	18 (3.5)	2 (2.8)	.234	.646
Medication at discharge^f^					
Verapamil	0 (0)	4 (0.8)	0 (0)	.999	
Digoxin	8 (6.8)	39 (7.5)	9 (12.5)	.999	.400
B-Blocker	91 (77.8)	424 (81.1)	61 (84.7)	.878	.528
Any antiarrhythmic	11 (9.4)	28 (5.4)	2 (2.8)	.264	.272
ECG parameters					
Heart rate, bpm	61 (53, 71)	61 (54, 70)	61 (54, 73)	.965	.902
PR-interval, ms	166 (154, 182)	195 (176, 216)	206 (184, 220)	<.001	<.001
Atrioventricular block	13 (10.9)	212 (40.5)	42 (58.3)	<.001	<.001
P-wave duration, ms	110 (105, 115)	135 (130, 145)	150 (140, 170)		
Ventricular conduction issue	20 (16.8)	124 (23.7)	14 (19.4)	.228	.999
Left ventricular hypertrophy	11 (9.6)	64 (12.7)	12 (17.4)	.858	.332

Data are given as median (interquartile range) for continuous variables and as frequency (percentage) for categorical variables.

*PIAB compared to normal P-wave; **AIAB compared to normal P-wave.

Data is missing in ^a^370, ^b^127, ^c^33, ^d^497, ^e^465, ^f^<10.

AF: atrial fibrillation; AIAB: advanced interatrial block; CHA2DS2-VASc: congestive heart failure, hypertension, age ≥75 (doubled), diabetes mellitus, and prior stroke, transient ischaemic attack or thromboembolism (doubled), vascular disease, age 65 to 74, sex category (female); CV: cardioversion; ECG: electrocardiogram; eGFR: estimated glomerular filtration rate; PIAB: partial interatrial block; TIA: transient ischaemic attack.

## Results

Interatrial blocks were a common finding in the study cohort: 524 (73.3%) had PIAB and 72 (10.1%) AIAB. Baseline characteristics grouped by interatrial block types are presented in Table 1. In univariate analyses, AIAB was associated with older age, hypertension, vascular disease and lower eGFR. Additionally, patients with IABs had longer PR intervals, and therefore, were more likely to have an AV-block. Ventricular hypertrophy or ventricular conduction issues were not associated with interatrial blocks.

We found a weak correlation (*r* = 0.195) between left atrial diameter and P-wave duration (*p* = .004). The majority of patients (78.0%) had at least mild left atrial enlargement. Patients with PIAB had significantly larger atria than those with normal P-wave or AIAB.

### Cardioversion efficacy

Data on CV failure, AF recurrence and ineffective CV are provided in Figure 3 and Table 2. CV failed in 63 (8.8%) of all cases, 16 of whom first had a successful CV, but the rhythm converted back to AF before hospital discharge. CV failure was equally common in both normal conduction (*n* = 8; 6.7%) and PIAB groups (*n* = 38; 7.3%), whereas patients with AIABhad significantly higher failure rates (*n* = 17; 23.6%; *p* = .002). In univariable and multivariable logistic regression analyses AIAB was the only significant predictor of CV failure.

**Table 2.** Multivariable regression identifying predictors of CV failure, AF recurrence and ineffective CV.

	CV failure			AF recurrence			Ineffective CV		
	OR	95% CI	*p*	OR	95% CI	*p*	OR	95% CI	*p*
AIAB	4.51	1.76–11.56	.002	2.93	1.43–5.99	.003	3.87	2.04–7.36	<.001
PIAB	1.10	0.49–2.47	.813	1.48	0.91–2.40	.111	1.45	0.93–2.26	.101
AV-block	0.83	0.47–1.46	.518	0.68	0.48–0.99	.041	0.69	0.50–0.97	.032
Any antiarrhythmic									
At CV	0.39	0.05–2.97	.367						
At discharge				1.75	0.87–3.54	.119	1.99	1.05–3.80	.036
Diabetes	0.73	0.33–1.63	.444	0.54	0.32–0.91	.021	0.55	0.35–0.89	.014
Hypertension	1.20	0.70–2.07	.509	1.32	0.93–1.87	.117	1.35	0.98–1.86	.067

PIAB and AIAB are compared against normal P-wave.

AF: atrial fibrillation; AIAB: advanced interatrial block; AV-block: atrioventricular block; CI: confidence interval; CV: cardioversion; OR: odds ratio; PIAB: partial interatrial block.

During the 30 days follow-up AF recurred in 209 out of 652 (29.2%) patients with a successful CV. When compared to the normal P-wave group (*n* = 29; 26.1%) those with AIAB were more likely to have recurrent AF (*n* = 25; 45.5%; *p* = .028). The difference was not statistically significant for PIAB (*n* = 155; 31.9%; *p* = .512). In univariable logistic regression analyses, only AIAB predicted recurrence. In the multivariable model, AIAB remained an independent predictor for AF recurrence, whereas AV-block or history of diabetes predicted maintaining SR.

Overall, CV was ineffective 272 out of 715 times (38.0%). In a multivariable analysis, AIAB remained the strongest predictor for ineffective CV, whereas PIAB did not gain significance. Additionally, the use of antiarrhythmic medication was an independent predictor for ineffective CV, however, individually tested the different types of antiarrhythmic agents were not significant. AV-block and history of diabetes remained independent predictors for maintaining SR.

### Cardioversion safety

Altogether 12 (1.7%) patients had episodes of asystole or bradycardia immediately after CV. There was no statistically significant difference between the occurrence of arrhythmic complications between AIAB patients (*n* = 3; 4.2%) and others (*n* = 9; 1.4%; *p* = .111). There were two ischaemic strokes and one mortality during the 30 days follow-up, all three events occurred to patients with PIAB (*p* = .580).

### Atrial stunning

We analyzed 50 extra ECGs to rule out the effect of atrial stunning in P-wave duration and shape. ECGs were collected 1.8 (SD 6.1) months post-CV on average. P-wave duration (delta 1.96 ms (SD 10.8), *p* = .200), number of biphasic P-waves (delta 0.078 (SD 0.891), *p* = .532), PTF (delta −7.00 mm*ms (SD 28.7), *p* = .090) or PR-interval (delta −3.12 ms (SD 18.7), *p* = .240) did not change significantly.

## Discussion

This study demonstrated that AIAB is a powerful predictor for ineffective CV as well as CV failure and AF recurrence separately. Strikingly, nearly two-thirds of CVs turned out ineffective in patients with AIAB – a significant increase compared to patients without such ECG findings. No other ECG marker of atrial cardiomyopathy proved to be a strong predictor. To the best of our knowledge, this study was the first to seek ECG parameters to assess both CV failure and AF recurrence in a large patient cohort. Our results regarding AIAB predicting AF recurrence were in line with previous small series studies [1,5,6], however, information regarding CV failure has not been previously published.

In this study, AIAB was associated with multiple known risk factors for atrial cardiomyopathy such as increasing age, hypertension, and vascular disease. These markers were not independent predictors for ineffective CV which highlights the fact that atrial remodelling is a multifactorial process. Changes in the atrial structure and conduction are, in turn, known to contribute to the AF burden. Strikingly diabetes, another risk factor for atrial myopathy and AF, did predict maintaining SR. Previously, diabetes has had mostly insignificant results regarding CV efficiency [1–4], but there are some exceptions, such as the FinCV-study regarding acute CVs [14]. Additionally, the presence of AV-block favoured effective CV. This is an interesting finding as AV-blocks were most common in the AIAB group and AV-blocks are another marker for atrial myopathy. The relationship between these blocks needs further investigation.

It is well known that older patients with enlarged left atria are less likely to achieve sinus rhythm [15,16]. In our cohort, the majority had atrial enlargement and those with PIAB seemed to have the largest diameters. Conversely, those with patients with normal P-wave and AIAB did not show a statistically significant difference. However, it is to be noted that the diameter data was available only on a third of patients and the frequency was especially low on patients with AIAB (*n* = 12, 15.8%).

Results also indicate that interatrial blocks appear to be more common amongst patients with AF than in the general population. We observed AIAB in every 10th patient undergoing CV for persistent AF and PIAB in 3 out of 4 patients whereas in the general population the frequencies were about 1% and 10%, respectively [17]. This underscores that any interatrial block is very common in this patient subset. Yet this ECG distinction makes a lot of sense as AIAB is strongly associated with new-onset AF [17–20]. Our study reinforced the association to AF given the 4-fold increase in ineffective CV odds ratio. Furthermore, AIAB is also strongly associated with other cardiovascular conditions such as hypertension and stroke [17–19,21].

### Clinical implications

So far attempts in identifying patients likely not to benefit from the CV of persistent AF have gained only modest success [2,4,7,15]. Some of these known predictors are usually assessed after the CV procedure, making them less useful in guiding whom to perform CV in the first place. Conversely, AIAB can often be assessed if an ECG has been taken before the persistent AF episode. Our findings indicate that in addition to clinical evaluation old ECGs should be reviewed when considering elective CVs for patients with persistent AF. This is an important observation as ECGs are recorded and stored electronically regularly as part of routine practice. Additionally, post-CV recordings should also be interpreted and AIAB findings noted to guide decision making in the future. AIAB appears to be drastically underdiagnosed even though it is an entity much more easily recognized than many other ECG abnormalities. Patients with AIAB are a subpopulation of AF patients with an increased risk of adverse events overall and therefore present an increased burden to the health care system.

The measurements in this study were conducted manually with no callipers to highlight the clinical availability of AIAB in the real-life setting, as opposed to some ECG studies relying on automated measuring and shape recognition.

### Limitations

The main limitation of this study is its retrospective nature. Nevertheless, the data were collected from electronic patient records where data on baseline, peri-CV, and outcomes are reported in detail. A structured case report form was used to ensure the uniformity of reporting. It should also be noted that the ECGs for CV failure patients were collected before or after the CV, while the rest were immediately post-CV. However, this study setting relies on the assumption that underlying atrial conduction pathologies are ongoing and non-regressing processes because underlying fibrosis and damage to the Bachmann bundle are thought to cause AIAB [9]. It is not known how fast AIABs may develop and therefore the results of this study are applicable only to those with a recent (within 60 months) SR recording. The stability of atrial ECG findings in short term (within a month of index CV) was also examined and found to be true by studying extra recordings in an attempt to rule out the possible effect of atrial stunning.

Another important limitation is that the detection of AF after hospital discharge was not based on continuous ECG recording (e.g. Holter or implantable rhythm recorders) but on ECG recordings during the 30 days follow-up visits and ECGs of symptomatic or otherwise detected AF episodes. Thus, the actual AF occurrence after the CV is likely to be higher as some of the asymptomatic AF episodes were most likely missed. The moderate sample size is another limitation of this analysis, and therefore, these findings should be viewed as hypothesis-generating.

Finally, this ECG-study excluded a third of the original patients due to non-available ECGs. Excluded patients were older and more likely hypertensive. The frequency of CV failure was higher for those excluded, however, this is a result from excluding patients with only AF films available. When all patients with available ECGs are considered, the failure rate rises to 15.1% in this cohort.

## Conclusions

Our findings demonstrate that AIAB independently predicted ineffective CV in patients with persistent AF. This study indicates that ECG will provide a powerful tool that helps clinicians identify patients with AF who benefit from elective CV, but the results need to be further validated.
